# Personality Styles of German-Speaking Psychotherapists Differ from a Norm, and Male Psychotherapists Differ from Their Female Colleagues

**DOI:** 10.3389/fpsyg.2017.00840

**Published:** 2017-05-24

**Authors:** Burkhard Peter, Eva Böbel, Maria Hagl, Mario Richter, Miguel Kazén

**Affiliations:** ^1^Department of Psychology, Ludwig-Maximilians-Universität MünchenMünchen, Germany; ^2^MEG-StiftungMünchen, Germany; ^3^Department of Psychology, Katholische Universität Eichstätt-IngolstadtEichstätt, Germany; ^4^Institute of Psychology, Universität OsnabrückOsnabrück, Germany

**Keywords:** Therapist variables, personality styles, PSDI, German-speaking psychotherapists, working alliance, therapist gender

## Abstract

Variables pertaining to the person of the psychotherapist have been neglected in psychotherapy research for some time. Concerning personality in particular, however, research has mostly focused on its relation with the psychotherapist’s choice of method, or differences between the various major therapy approaches. That is, psychotherapists were compared to each other without specifying how exactly psychotherapists are in comparison to “ordinary people.” We wanted to know: Are there specific personality styles that distinguish psychotherapists from the norm? A sample of 1,027 psychotherapists from Germany, Austria, and Switzerland filled out the short version of the Personality Style and Disorder Inventory (PSDI-S) via online survey. The PSDI-S is a self-report questionnaire that assesses 14 personality styles, partly related to the non-pathological equivalents of classifiable personality disorders. The psychotherapists were compared to a normative sample of 3,392 people of different professions. The results could be divided into three groups: (1) Large differences in four personality styles that might contribute to relationship skills and may enable psychotherapists to put their own personal opinion aside, show empathy and appreciation, open themselves to the emotional experience of the patient, and provide a trusting relationship. (2) Moderate differences in seven personality styles that are equally indicative of the professional social skills of the psychotherapists, i.e., they were neither submissive nor passive, not excessively helpful, but also not too self-assertive. (3) Hardly any or no differences regarding a charming (histrionic) style, optimism, and conscientiousness. Gender-specific results revealed that male psychotherapists differed from their female colleagues, but they did so differently than men and women in the normative sample do. The main limitations were that we relied on self-report and did not statistically control for gender, age, and education, when comparing to the norm. As a conclusion, German-speaking psychotherapists show personality styles that we interpret as functional for psychotherapeutic practice but this needs corroboration from studies that use different methods and measures.

## Introduction

Therapist variables have been a topic of ongoing interest in psychotherapy research for more than half a century. Important groundwork was laid by pioneers like Rogers (1957) and Frank (1961). Both of them paid special attention to personal characteristics of psychotherapists and their impact on the therapeutic alliance, which has been identified as one of the strongest and most consistent predictors of outcome in various psychotherapeutic approaches (Orlinsky et al., 2004; Norcross and Wampold, 2011). Reviews and meta-analyses report that it accounts for 5–10% of therapeutic success in controlled clinical trials and up to 17% in health care research (Willutzki et al., 2013; Green et al., 2014; Firth et al., 2015). The personality of psychotherapists, however, represents only one part of the broader spectrum of the therapist variables that Beutler et al. (1994) categorized into four quadrants: (1) Observable traits like gender or age, (2) inferred traits like personality or values, (3) observable states like professional background or interventions, and (4) inferred states like relationships or expectancies. This article contributes to the first two quadrants: the personality of psychotherapists and whether it is related to gender, and if so, how. But first, we want to give a more general overview of the research on this topic.

Beutler et al. (2004), as well as already Bergin (1997) and Garfield (1997), lamented that the therapist variables did not have play an adequate part in efficacy studies. Klug et al. (2008) even bluntly posed the question: “The therapist variable. Is it still an unknown factor?” Two comprehensive reviews of Beutler et al. (1994, 2004) were generally concerned with the therapist variables but they did not present much data on personality styles of psychotherapists, and Orlinsky et al. (2015) in their most recent review neglected this topic of personality styles completely when discussing the relationship qualities of the therapists.

Many other aspects of the therapist variables have been of interest to investigators: Studies researched factors like connections between therapist’s personal orientation, therapeutic style, and chosen therapy approach (Schacht and Black, 1985; Arthur, 2000, 2001; Poznanski and McLennan, 2003; Castañeiras et al., 2006; Klug et al., 2008; Heffler and Sandell, 2009; Taubner et al., 2010). The results were not completely consistent, partially due to the use of different measures. Factors involved in the self-selection process in choosing therapeutic training have also been given attention (Taubner et al., 2014). There is evidence that the suitability of an applied therapy to a therapist’s personality affects satisfaction with her/his training and work (Topolinski and Hertel, 2007; Vangermain and Brauchle, 2013; Taubner et al., 2014). Above all, there seem to be correlations between therapists’ personalities and the choice of therapy approach (Tremblay et al., 1986; Arthur, 2001; Poznanski and McLennan, 2003; Ogunfowora and Drapeau, 2008; Boswell et al., 2009; Buckman and Barker, 2010), which are not only reflected in the therapist’s self-ratings but also by the ratings of others (Keinan et al., 1989). Such choices and attitudes can change not only in the course of training but even more importantly, in the course of professional life. The fit between the personalities of the patient and the therapist (Taber et al., 2011) and other topics like the patient’s symptom severity on outcome and alliance (Dinger et al., 2016) have been investigated. In the most recent decades the relationship between therapists’ personality characteristics, working alliance and outcome of psychotherapy also has been studied and reviewed (Ackerman and Hilsenroth, 2001, 2003; Orlinsky et al., 2004; Norcross and Wampold, 2011). In several studies early parental relations and attachment styles of psychotherapists were correlated with therapeutic alliance. For example, Hilliard et al. (2000) found that negative experiences in early parental relations were associated – as expected – with worse therapeutic relationship but – unexpectedly – seen only from the perspective of the therapist, but not from the perspective of the patient or an independent rater. A similar result, that there is no influence of therapists’ interpersonal problems on the patient-rated alliance and therapy outcome, was reported in a first study conducted by Dinger et al. (2007). In a later attachment study, however, Dinger et al. (2009) revealed that therapists’ attachment security was not related to alliance development in general, but a higher “reoccupation” attachment style in therapists (i.e., being insecure of significant others’ feelings or tending to cling to and to control others) predicted worse therapist-patient alliances. Similarly, Hersoug et al. (2009) found a worse patient- and therapist-rated alliance if the therapist was “cold” or “detached” in private life, whereas therapists’ representation of a more caring mother was associated with higher patient ratings of the therapeutic alliance. Schauenburg et al. (2010), on the contrary, could not replicate these findings of therapists’ attachment styles in private life and patient-rated-alliance, but found that a higher attachment security of the therapist facilitated better alliance among severely impaired patients. Heinonen and Orlinsky (2013) compared therapists’ relational styles at work and at home (i.e., toward patients and close personal relationships) and found a remarkable reverse relation: stereotypically neutral psychoanalysts were privately warmer, friendlier and more nurturing, while cognitive-behavioral therapists were less directive, less challenging, and more intuitive in close personal relationships, than they were with clients. Nissen-Lie et al. (2010) showed in a large-scale study that therapists’ negative personal reaction to patients (such as hostility or frustration) predicted – as expected – worse patient-rated alliances. In contrast, therapists’ humbleness and sensitivity regarding his/her relational skills predicted better patient-rated alliances – in sharp contrast to therapists with greater self-reported “advanced” relational skills, which predicted worse alliances. Finally it was shown that therapists differ in effectiveness with regard to patient’s symptom severity, and in the degree of the association between positive alliance and therapeutic outcome (Dinger et al., 2007, 2008), or, in general, that therapist variability in the alliance seems much more predictive for outcome than patient variability: a high rated alliance correlated with better outcome and vice versa (Baldwin et al., 2007; Del Re et al., 2012).

Taken together, recent research has revealed that therapists who are perceived by their patients as being (1) warm, accepting, engaged, empathic, and responsive, as well as (2) open, flexible, and respectful seem to establish better working alliances (Horvath and Bedi, 2002; Horvath et al., 2011) which in consequence leads to better outcomes (Orlinsky et al., 2004; Norcross and Wampold, 2011). Considering the fact that such personality aspects of psychotherapists are helpful for doing psychotherapy it is surprising that there are only few studies that took this issue into account and sought to measure personality aspects of psychotherapists. For some of these studies, special rating scales were designed (Keinan et al., 1989; Sandell et al., 2004; Orlinsky and Rønnestad, 2005). In a few studies, standard personality inventories like the NEO-PI-R (aka NEO-FFI, the “Big Five”; see **Table 1**) (Costa and McCrae, 1992) were used. Some have shown that psychotherapists (practitioners or trainees) with a psychodynamic orientation exhibit greater *openness to experience* compared to those with a cognitive-behavioral orientation (Poznanski and McLennan, 2003; Topolinski and Hertel, 2007; Boswell et al., 2009; Buckman and Barker, 2010; Taubner et al., 2014), but results for the other NEO-PI-R dimensions were less conclusive.

**Table 1:** The Big Five personality traits of the NEO-PI-R (Costa and McCrae, 1992).

Traits	Characteristics
Openness to experience	Inventive/curious vs. consistent/cautious
Conscientiousness	Efficient/organized vs. easy-going/careless
Extraversion	Outgoing/energetic vs. solitary/reserved
Agreeableness	Friendly/compassionate vs. analytical/detached
Neuroticism	Sensitive/nervous vs. secure/confident

In all of these studies, psychotherapists were compared to each other without specifying how exactly psychotherapists are, for example, in comparison to “ordinary people.” To our knowledge, only one of these studies compared psychotherapists’ personality scores with a normative sample: In 46 US-American graduate student therapists in-training, Boswell et al. (2009) found participants’ openness and neuroticism scores to be in the high range, while extraversion, agreeableness, and conscientiousness were in the average range. Only few studies have controlled for gender differences (Ogunfowora and Drapeau, 2008) or at least reported them. Using the Millon Index of Personality Styles (MIPS) (Millon et al., 1994), Arthur (2000) found 9 significant gender differences in 12 scales in a sample of 247 psychotherapists from the UK. Researching the Big Five in 162 Finnish substance abuse therapists, Saarnio (2010) found that the 119 females were friendlier and more open to experience than their 43 male colleagues. However, this sample included only 7 psychologists or physicians; the others were nurses, social workers or counselors.

Finally, few German studies have explicitly addressed the matter of personality factors (e.g., Topolinski and Hertel, 2007; Taubner et al., 2014). In our previous research (Peter et al., 2012) we assessed 203 German-speaking practitioners of hypnosis or hypnotherapy (49% were psychological or medical psychotherapists) with the Personality Style and Disorder Inventory (PSDI) of Kuhl and Kazén (2009), which measures personality styles that partly hint at personality disorders when expressed in the extreme (**Table 2**). Hypnosis practitioners differed significantly from a normative sample of the PSDI in all but one of the scales: They were below the norm in 9 of 14 personality styles, and above the norm in 4 styles. Because of the “hypnosis” context, we could not generalize these differences to psychotherapists regardless of their interventional approach. So, despite the study of Boswell et al. (2009) and ours, we still do not have enough data on whether or how, respectively, psychotherapists differ from the norm. This seems to us to be a question of general interest regarding the importance of therapist variables for therapy outcome.

**Table 2:** The 14 scales of the Personality Styles and Disorders Inventory (PSDI/PSDI-S) by Kuhl and Kazén (2009).

PSDI-scale^a^	Example
PN: willful/**paranoid**	“Most people mean well” (negatively coded)
BL: spontaneous/**borderline**	“My feelings often change abruptly and impulsively”
SZ: reserved/**schizoid**	“I always keep my distance to other people”
NA: ambitious/**narcissistic**	“The idea of being a famous personality appeals to me”
AB: loyal/**dependent**	“I need a lot of love and acceptance”
NT: critical/negativistic	“I have frequently been persecuted by bad luck”
ST: intuitive/**schizotypal**	“There are supernatural forces”
SL: unselfish/self-sacrificing	“I am more concerned with other people’s worries than my own needs”
SU: self-critical/**avoidant**	“Criticism hurts me quicker than it does to others”
DP: passive/depressive	“I often feel low and feeble”
AS: assertive/**antisocial**	“If people turn against me I can get them down”
HI: charming/**histrionic**	“My good moods are very contagious to others”
RH: optimistic/rhapsodic	“I am an invincible optimist”
ZW: conscientious/**compulsive**	“Consistency and firm principles define my life”

*^a^DSM-5 or ICD-10 equivalents are in bold print.*

The current study, an online survey, is, to the best of our knowledge, the first in which practicing psychotherapists were researched by means of the PSDI-S, not only in Germany but also in the German-speaking countries of Switzerland and Austria, and regardless of their principal intervention approach. It was necessary, as a preliminary analysis, to check whether the three country-specific samples could be treated as a single sample. The main research questions of this explorative study was: Do psychotherapists differ from a normative sample? As gender differences in psychotherapists were seldom researched before, the second research question was: Do male psychotherapists differ from their female colleagues?

In light of our previous findings, we expected psychotherapists to differ considerably from the mean value of a norm derived from the PSDI-S’ normalization sample. However, as our previous research mostly focused on practitioners of hypnosis or hypnotherapy, we refrained from formulating hypothesis regarding the psychotherapists’ overall personality pattern, gender differences, or specific PSDI-S scales.

## Materials and Methods

### Sample

The sample consisted of 1,027 psychotherapists from Germany, Austria, and Switzerland. Within those three countries, approximately, 4,600 psychotherapeutic practitioners were initially contacted via e-mail (830 from Switzerland, 610 from Austria, and 3,160 from Germany), yielding a rate of approximately 22% responses. Of those respondents, 628 (61.1%) were from Germany, 285 (27.8%) were from Switzerland, and 114 (11.1%) were from Austria. More than two-thirds of the participants (71.4%) were female, which corresponds roughly to the proportion of female practitioners in these countries.^1^ The mean age was 53.5 years (*SD* = 10.6) and the duration of professional practice on average 19.34 years (*SD* = 10.75). A little less than half of the participants (42.2%) have been professionally active for more than 20 years; 22 were no more active. The majority of respondents (79.9%) were psychologists, while the proportion of physicians was low (4.7%). Overall, 158 (15.4%) respondents had an educational background that was not based in psychology or medicine: Germany *n* = 61 (9.7%), Switzerland *n* = 33 (11.56%), Austria *n* = 64 (56.14%).^2^

### Measures

To assess the personality styles, the short form of the PSDI of Kuhl and Kazén (2009) was used. The PSDI (Kuhl and Kazén, 2009) is a self-rating questionnaire that assesses the relative manifestation of personality styles, partly related to the non-pathological equivalents of the personality disorders of the DSM-5 (American Psychiatric Association, 2013) and the ICD-10 (World Health Organization, 1992). Its long version consists of 140 items that are divided into 14 scales (**Table 2**), each with 10 items. We used the short version (PSDI-S) consisting of 56 items (4 items per scale) because of high dropout rates in our former studies using the long version. Each item is answered on a four-point Likert scale (from “*strongly disagree*” to “*strongly agree*”), resulting in 0–12 points per scale which are transformed into *T*-values. Kuhl and Kazén (2009) emphasized that *T*-values above or below one standard deviation of the mean *T*-value of 50 (i.e., outside the range of 40–60) at an individual level can suggest the presence of a personality disorder (but not ascertain it, unless a proper diagnosis is carried out). The normalization sample of the PSDI-S consisted of 3,392 participants (1,763 women and 1,629 men) between the ages 12–82, who had different occupations (students, managers, regular employees, and homemakers; unpublished data provided by Kazén, 2017). The PSDI-S is standardized, provides objective procedures and analyses, and mostly has satisfactory reliability (Cronbach’s α = 0.64–0.79). The validity of the PSDI was established in different studies, where it showed medium to strong correlations with personality inventories such as the Big Five and the 16 PF-R (Sixteen Personality Factor Questionnaire) (Cattell et al., 1993). **Table 2** presents the individual personality styles and the corresponding pathological manifestations that are assessed by means of the PSDI/PSDI-S.

### Recruitment and Data Collection

Data collection took place between April 29th and June 5th of 2015 using SoSci Survey software. The e-mail addresses of the individual psychotherapists were obtained from the search portals of three websites listing mostly psychological psychotherapists, specifically from the German Association of Psychotherapists ^3^, the Austrian Ministry of Health ^4^, and the Federation of Swiss Psychologists^5^ (Search specified with “German language” and “psychotherapy”).

The recipients were invited to the survey as follows:

As part of a project on personality styles of people in the helping professions (in comparison to those in other professions), we are in need of information from psychotherapists who are or have been in practice, especially since only students have been examined to date. Therefore, we kindly ask you to click on […] and answer anonymously the 56 statements from the Personality Style and Disorder Inventory (PSDI; Kuhl & Kazén). Because the questions should be answered quickly and spontaneously, it will only require about 5 min to complete. Of course, your answers are completely anonymous.

Prior to answering the 56 items of the PSDI-S, some data pertaining to demographics and professional practice were obtained.

### Data Analysis

The data collected with SoSci Survey were loaded directly into SPSS (Version 23). The confidence intervals for effect sizes were obtained with the statistical software R (Version 3.2.2). Hypotheses were tested using *t*-tests or one-way analyses of variance (ANOVAs) depending on the number of populations that were compared. None of the PSDI scales was normally distributed. However, as both *t*-test and one-way ANOVA are considered to be robust against violations of the assumption of normality, especially in large samples, we chose to refrain from using non-parametric tests for two reasons: Firstly, for having more power to detect existing differences and secondly, for having the possibility to compute confidence intervals that allow us to gauge the magnitude of these differences. Levene tests were used to assess homogeneity of variances. In the event of heterogeneity of variances, the *t*-test for independent samples (as well as the ANOVA) was replaced by the Welch test. Because of multiple comparisons the threshold for significance was set after Bonferroni correction at *p* = 0.0018. ^6^

## Results

### Differences Regarding Country

The first question focused on whether there were differences in personality styles among the three German-speaking nationalities. This was done to determine whether those surveyed did not differ too much to be used as a single, pooled sample. This question was assessed using one-way ANOVAs with three levels. The results revealed that in one PSDI scale at least one country’s group significantly differed from the two other countries, namely concerning the intuitive ST style, *F*(2, 279.39) = 19.15, *p* < 0.0001 (with unequal variances).

The Games-Howell *post hoc* analysis of the comparison of the countries revealed that German psychotherapists were, on average, less intuitive (ST) than their Austrian and Swiss colleagues, with small to medium effects in both cases (**Table 3**). No difference was found in the intuitive (ST) style between Austrian and Swiss Psychotherapists. Only 3.9% of the variance accounted for the difference between nationalities (German, Austrian, and Swiss) for the intuitive (ST) style (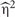
=0.039).

**Table 3:** Intuitive (ST) personality style (*T*-scores) regarding country (*post hoc*-analysis).

Country	*M*	*SD*	*p*	*d*	*CI*_0.95_
Austria	47.49	8.23			
Austria vs. Germany			0.003	–0.38	–0.58; -0.18
Germany	44.79	7.28			
Swiss vs. Germany			<0.0001	–0.44	–0.58; -0.30
Swiss	48.11	8.88			

*For *post hoc*-analysis the Games-Howell test was used that already adjusts for Type I Error, so no additional Bonferroni correction was needed.*

### Differences from the Norm

Single sample *t*-tests were used to determine whether psychotherapists differed from the mean value of the norm as defined in the normative sample (*N* = 3,392) according to personality styles, which they actually did in 12 of the 14 personality styles. The mean *T*-scores and standard deviations of the personality styles, the results of the *t*-tests and corresponding *p*-values, and Cohen’s *d* together with confidence intervals can be found in **Table 4**.

**Table 4:** Comparison of the 14 personality styles (*T*-scores) of the psychotherapists (*N* = 1,027) and the mean value of the norm (*T* = 50).

Personality style	*M*	*SD*	*t*(1026)	*d*	*CI*_0.95_
Willful (PN)	42.18	7.96	–31.48^∗∗∗^	–0.98	[-1.06; -0.91]
Spontaneous (BL)	43.10	5.20	–42.56^∗∗∗^	–1.3	[-1.41; -1.24]
Reserved (SZ)	43.13	9.00	–24.45^∗∗∗^	–0.76	[-0.83; -0.69]
Ambitious (NA)	43.21	7.20	–30.24^∗∗∗^	–0.94	[-1.02; -0.87]
Loyal (AB)	44.77	8.01	–20.90^∗∗∗^	–0.65	[-0.72; -0.58]
Critical (NT)	45.43	7.00	–20.92^∗∗∗^	–0.65	[-0.72; -0.59]
Intuitive (ST)	45.95	8.00	–16.18^∗∗∗^	–0.5	[-0.57; -0.44]
Unselfish (SL)	46.01	8.42	–15.16^∗∗∗^	–0.47	[-0.54; -0.41]
Self-critical (SU)	46.73	7.59	–13.82^∗∗∗^	–0.43	[-0.49; -0.37]
Passive (DP)	47.08	6.85	–13.66^∗∗∗^	–0.43	[-0.49; -0.36]
Assertive (AS)	47.18	7.68	–11.75^∗∗∗^	–0.37	[-0.43; -0.30]
Charming (HI)	48.70	8.51	–4.91^∗∗∗^	–0.15	[-0.21; -0.09]
Optimistic (RH)	49.97	8.73	–0.82	–0.03	[-0.09; -0.04]
Conscientious (ZW)	49.85	8.33	–0.58	–0.02	[-0.08; -0.04]

*^∗∗∗^*p* < 0.001.*

**FIGURE 1:**
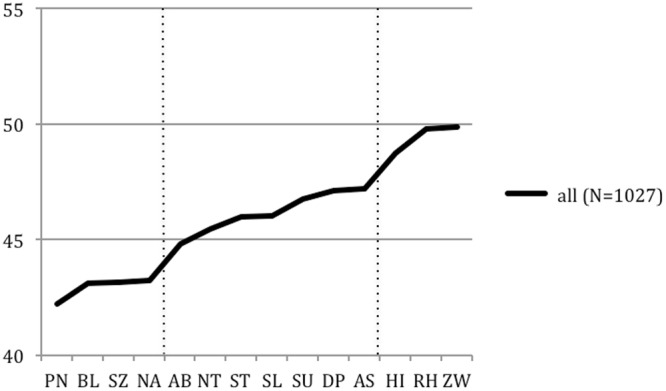
**Personality profile (*T*-scores) of psychotherapists (*N* = 1,027) in comparison to the normative sample (*N* = 3,392).** The average scores of the psychotherapists were in the normal range of 40 and 60, but consistently below the mean average *T*-value of 50.

When comparing personality styles of psychotherapists to the norm, large effect sizes were found for the willful (PN), spontaneous (BN), reserved (SZ), and ambitious (NA) styles. Small to no differences were found in the charming (HI), and no differences were found in the optimistic (RH) and the conscientious (ZW) styles. All the other differences had moderate effect sizes. Among all of the effects psychotherapists scored lower on each personality style compared to the mean value of the normative sample, as they all had *T-*scores lower than 50 (**Figure 1**).

### Differences Regarding Gender

To determine whether male and female psychotherapists differed among the assessed personality styles, 14 *t*-tests were conducted. Eight of the 14 comparisons indicated significant gender differences, namely concerning the reserved (SZ), ambitious (NA), intuitive (ST), unselfish (SL), self-critical (SU), assertive (AS), charming (HI), and optimistic (RH) style. The sign of the *T*-scores should be interpreted based on the way gender was coded: positive *T*-scores mean that females have greater *T*-scores compared to males, and vice-versa. The mean differences, *t*-values, Cohen’s *d*, and confidence intervals for the effect sizes of eight personality styles are presented in **Table 5**.

**Table 5:** Comparison of male and female psychotherapists among eight personality styles (*T*-scores).

Personality Style	*M_w_*-*M_m_*	*t*(1025)	*d*	*CI*_0.95_
Reserved (SZ)	–3.06	–4.98ˆ***	–0.34	[-0.48; -0.21]
Ambitious (NA)	–2.00	–4.06ˆ***	–0.28	[-0.42; -0.14]
Intuitive (ST)	1.81	3.56ˆ***	0.22	[0.09; 0.36]
Unselfish (SL)	2.10	3.63ˆ***	0.25	[0.11; 0.39]
Self-critical (SU)	1.92	3.81ˆ***	0.25	[0.12; 0.39]
Assertive (AS)	–2.06	–3.91ˆ***	–0.26	[-0.41; -0.13]
Charming (HI)	3.15	5.44ˆ***	0.38	[0.23; 0.51]
Optimistic (RH)	2.33	3.89ˆ***	0.27	[0.13; 0.40]

*The assumption of equal variances was not met for the assertive (AS), *F*(1, 1,025) = 4.67, *p* < 0.05, and reserved (SZ) styles, *F*(1, 1,025) = 11.19, *p* < 0.05. ^∗∗∗^*p* < 0.001.*

**FIGURE 2:**
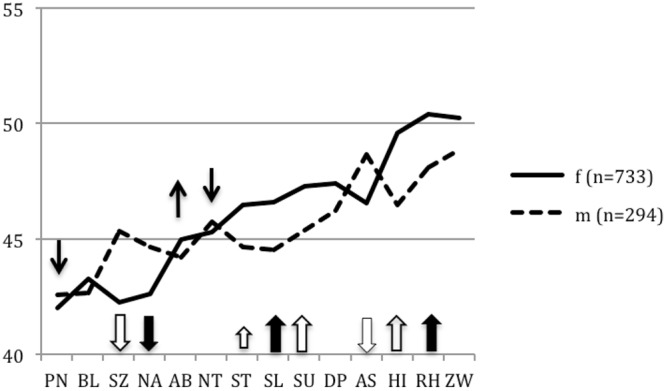
**Personality profiles (*T*-scores) of psychotherapists according to gender (female: *n* = 733; male: *n* = 294) in comparison to the norm (Mean: 50; Normal range: 40–60).** The “empty” arrows point to the five gender differences that appeared in both the normative sample and in the present study. If one considers the five gender differences as general ones, that leaves the three bolded arrows that point to the NA, SL, and RH personality styles, in which either male psychotherapists differed from their female counterparts (SL, RH), or vice versa (NA). Moreover, there are three gender differences that are significant in the normative sample but not among the psychotherapists, namely the PN, AB, and NT styles. They are indicated by the regular arrows.

As shown in **Table 5**, seven of the personality styles had a small to medium effect, and one (intuitive, ST style) showed nearly no or a small effect. **Figure 2** displays the gender differences among personality profiles.

The data show that female psychotherapists were less reserved (SZ), less ambitious (NA), and less assertive (AS) than their male colleagues. On the other hand, they were more unselfish (SL), more self-critical (SU), more charming (HI), more optimistic (RH), and somewhat more intuitive (ST).

As gender-specific differences were found in an earlier psychometric investigation by Kuhl and Kazén (2009) using the long form of the PSDI, they were compared with the gender-specific differences in our current sample of psychotherapists. We wanted to see if any differences according to gender were to be found exclusively in male and female psychotherapists. For that aim, the raw mean differences of the normative sample of the PSDI long version (*N* = 1,903) and those of our psychotherapists (*N* = 1,027) were analyzed with respect to the effect sizes for these differences and their respective confidence intervals (**Table 6**). Five of the personality styles, namely the SZ, ST, SU, AS and HI styles, hardly differed concerning gender differences (see the blank arrows in **Figure 2**). Because the confidence intervals overlap each other very strongly, we assume that there are no significant differences between the means of the two samples (the slightly smaller confidence intervals of the normative sample are due to its sample size being nearly twice as large). However, significant differences were found in the ambitious (NA), unselfish (SL), and optimistic (RH) styles among the sample of psychotherapists, while these styles were not significantly different in the normative sample. So, of the eight styles reported above in which female psychotherapists differed from their male counterparts, only three are relevant for this investigation: NA, SL, and RH. Female psychotherapists were found to be conspicuously less ambitious (NA) but more unselfish (SL) and more optimistic (RH) than their male colleagues (see the bold arrows in **Figure 2**). On the other hand, some of the gender differences found in the normative sample were not significant in our sample of psychotherapists, namely the willful (PN) and loyal (AB) styles, but most importantly the critical style (NT), for which the confidence intervals did not overlap at all (see the narrow arrows above the profile lines in **Figure 2**).

**Table 6:** Comparison of the gender differences and confidence intervals in a normative sample of the long version of PSDI from Kuhl and Kazén (2009) with the current sample of psychotherapists (raw scores each).

Personality style	*M_w_*-*M_m_* (normative sample, *N* = 1,903)	*M_w_*-*M_m_* (our sample, *N* = 1,027)	*CI*_0.95_ for δ	*CI*_0.95_ for δ
Willful (PN)	–0.91ˆ***	–0.12	[-0.29; -0.20] Small effect	[-0.21; 0.07] No or small effect
Spontaneous (BL)	–0.02	0.18	[-0.09; 0.09] No effect	[-0.01; 0.26] No or small effect
*Reserved (SZ)*	–*2.13^∗∗∗^*	–*0.7^∗∗∗^*	[-0.53; -0.35] Medium effect	[-0.48; -0.21] Small to medium effect
**Ambitious (NA)**	0.23	**-0.53^∗∗∗^**	[-0.05; 0.13] No or small effect	[-0.42; -0.14] Small to medium effect
Loyal (AB)	0.92ˆ***	0.22	[0.08; 0.26] No or small effect	[-0.04; 0.23] No or small effect
Critical (NT)	–2.27ˆ***	–0.11	[-0.58; -0.40] Medium effect	[-0.20; 0.07] No or small effect
*Intuitive (ST)*	*1.50^∗∗∗^*	*0.53^∗∗∗^*	[0.16; 0.35] Small effect	[0.09; 0.36] Small effect
**Unselfish (SL)**	–0.08	**0.51^∗∗∗^**	[-0.10; 0.08] No or small effect	[0.11; 0.39] Small to medium effect
*Self-critical (SU)*	*1.08*	*0.50*	[0.11; 0.30] Small to medium effect	[0.12; 0.39] Small to medium effect
Passive (DP)	–0.64	0.31	[-0.22; -0.04] No or small effect	[0.04; 0.31] No or small effect
*Assertive (AS)*	–*2.46^∗∗∗^*	*-0.53^∗∗∗^*	[-0.55; -0.36] Medium effect	[-0.41; -0.13] Small to medium effect
*Charming (HI)*	*1.89^∗∗∗^*	*0.82^∗∗∗^*	[0.26; 0.44] Small to medium effect	[0.23; 0.51] Small to medium effect
**Optimistic (RH)**	0.76	**0.63^∗∗∗^**	[0.05; 0.23] No or small effect	[0.13; 0.40] Small to medium effect
Conscientious (ZW)	–0.52	0.36	[-0.18; 0.00] No or small effect	[0.03; 0.30] No or small effect

*Asterisks point out significant mean differences; mean differences significant in both samples are printed in italics; mean differences significant in only the psychotherapist sample are printed in bold. The somewhat more precise estimate of the unknown population effect based on the normative data from Kuhl and Kazén (2009) is due to their sample being almost twice as large. ^∗∗∗^*p* < 0.001.*

## Discussion

As part of a project that investigated the therapist variable, 1,027 psychotherapists from Germany, Austria, and Switzerland took part in a 2015 investigation of personality profiles using the abbreviated version of the Personality Style and Disorder Inventory (PSDI-S; Kuhl and Kazén, 2009). Due to the nature of the websites used for the online survey of the study, mainly psychologists and few medical doctors were recruited. The main question of interest was if and how they differed from a norm, as established in a normative sample of 3,392 participants. Further, we were interested in possible gender differences.

In order to illustrate the different personality styles, we include, in this Discussion section, the names and descriptions of the respective personality disorders. For example, instead of merely referring to a personality style as “willful,” we refer to it as “willful/paranoid (PN).” This is only for illustrative purposes. The additional nomenclatures are the commonly used terms in the clinical field when personality styles are so extreme that they reach the point of pathology. The mean scores of those sampled were within the range established by Kuhl and Kazén (2009) as normal expressions of personality styles. In fact, 12 of 14 styles were significantly below the norm, four with a large effect; that is, they scored far from the respective personality disorders. This is important to bear in mind since a cliché about psychotherapists is that they, or at least many of them, have mental problems (Schmidbauer, 1977; Jaeggi, 2004; von Sydow, 2007, 2014).

As a preliminary analysis, we examined whether the three country-specific samples could be treated as a single sample. Only one country-specific difference was found among the 14 styles: German psychotherapists were significantly less intuitive/schizotypal (ST) than those in Austria and Switzerland. Because this effect was small to moderate, and there were no significant differences regarding country in the 13 other scales of the PSDI-S, we dismissed country as a relevant factor and analyzed the sample as a whole.

The first of our two primary research questions was whether the 1,027 psychotherapists differed in their personality profiles from the norm. Although their average personality profile was within the normal range of 40 and 60, they were significantly below the mean of 50 (*T*-scores) in 12 of 14 personality styles. This suggests that the characterization of our psychotherapists is made up by negations, so we will describe *how they are not*. For this characterization, we will partly use the terminology of Kuhl and Kazén (2009). Overall, the personality styles in which our sample differed from the norm can be split into three groups: those with large, medium, or small to no effect sizes (**Figure 1**).

### Differences with Large Effects

The psychotherapists showed very low levels of willful/paranoid (PN), spontaneous/borderline (BL), reserved/schizoid (SZ), and ambitious/narcissistic (NA) styles. That is, they differed from the mean value of the norm with a large effect.

– The willful/paranoid style (PN) describes those who are eager to guard themselves from others. In the extreme form, they interpret other people’s actions as insulting or even threatening.

This is the exact opposite of how, in our opinion, psychotherapists should conduct themselves. The primary and basic attitude of a psychotherapist should be to open up, be interested and tune in into the patient’s special worldview without judgement, that is, with an attitude of unconditional positive regard (Rogers, 1957). Opening up and not focusing on one’s own sensitivities is also quite the opposite of the NA style:

– The ambitious/narcissistic style (NA) describes people who perceive themselves as something special. Because they are wrapped up in their own grandeur, they show a lack of empathy.

Such a style does not apply to our psychotherapists either. They put their individuality inconspicuously into the background and show empathy, appreciation, and warmth for others, which is also in direct opposition of the reserved/schizoid SZ style:

– Those with the reserved/schizoid style (SZ) are people with an impaired emotional experience and intensity of expression; they show a sober dispassion and indifference to social relationships.

Psychotherapists, however, need to be open for the emotional experiences of their patients and be able to sympathize and to provide empathy appropriately, while maintaining a proper but not extreme professional emotional distance. This obviously applies to our surveyed psychotherapists:

– The spontaneous/borderline style (BL) is characterized by an intensive yet unstable emotionality. The instability refers to one’s own identity and especially to interpersonal relationships.

Quite the contrary should take place in psychotherapy: emotional encounters with the patients’ feelings should be moderate and *stable*. The personal expression of psychotherapists should not be extreme or volatile in order to provide the patient with a safe, warm, and trusting relationship. This attitude was found among our psychotherapists.

As we do not have data on the effectiveness of our psychotherapists, we can only make inferences on the possible meaning of our results by aligning them with the findings from other research. In this light we interpret the lower scores in these four personality styles as *necessary* for the therapeutic or working alliance. The interaction between therapist and patient, the working alliance, is considered to be crucial to psychotherapy outcome. Baldwin et al. (2007) found that psychotherapists’ variability in the alliance predicted the outcome of psychotherapy and concluded that therapists should receive training to develop and maintain strong alliances. The impact of the alliance on outcome was also found by Dinger et al. (2007) and confirmed by Dinger et al. (2008). Also, psychotherapists’ negative personal reactions to patients like low empathy, hostility, frustration or lack of tolerance – which could be understood as manifestations of the willful/paranoid (PN), the reserved/schizoid (SZ), and the spontaneous/borderline (BL) styles – predicted worse patient-rated alliances (Nissen-Lie et al., 2010), a finding, that was fairly stable in six measurements in the course of psychotherapy. Another finding of these researchers may relate to our result concerning the very low value in the ambitious/narcissistic style (NA): A kind of “professional self-doubt” (i.e., humbleness and caution and sensitivity to patients) predicted better patient-rated alliance in the study of Nissen-Lie et al. (2010), while greater self-reported “advanced relational skills” predicted worse alliances. Even an average amount of narcissism might result in a too self-centered stance that would obstruct a psychotherapist’s exclusive focus on the patient, which in turn would negatively impact the working alliance.

These four personality styles in our psychotherapist sample seem to represent a patient-centered therapeutic attitude in accordance with two of Rogers (1957) conditions: empathetic attention and unconditional positive regard. (The third of these so called Rogerian conditions, congruence, will be discussed below.) Zuroff et al. (2010) found that between-therapist variability in patients’ perceptions of the Rogerian conditions was related to overall clinical outcome, which also points to the importance of expressing these attitudes.

One could interpret these fundamental aspects of the psychotherapist’s personality also within the context of the NEO-FFI factors (Costa and McCrae, 1992), as three of these styles, willful/paranoid (PN), reserved/schizoid (SZ), and spontaneous/borderline (BL), were found to correlate negatively with extraversion, with the first two also correlated negatively with agreeableness (Kuhl and Kazén, 2009). Extraversion and agreeableness certainly contribute to the relationship skills of psychotherapists. Taking into consideration those personality styles that show large differences from the norm, we can conclude that our psychotherapists are highly capable of building and maintaining a solid therapeutic alliance.

### Differences with Medium Effects

There was a *second group* of personality styles, in which the psychotherapists differed from the normative sample, but to a *moderate degree, i.e., with medium effects.* This group consists of the AB, NT, ST, SL, SU, DP, AS, and also the HI scales, the latter to a smaller degree (which is why we put it to the third group; see **Figure 1**). In comparison to the normative sample, psychotherapists were less loyal/dependent (AB), critical/negativistic (NT), intuitive/schizotypal (ST), unselfish/self-sacrificing (SL), self-critical/avoidant (SU), passive/depressive (DP), and assertive/antisocial (AS). We will now examine whether these personality styles also contribute to aims and processes of psychotherapy, and, if so, which ones. Again, our argumentation is speculative, interpreting these results in the light of psychotherapy research and theory.

Our psychotherapists showed low score in the loyal/dependent (AB) personality style. One could assume that they are not subservient to or even dependent on their patients. The lowly expressed AB style indicates that they are able to stay congruent with themselves as professional psychotherapists while being with the patient. This would perfectly fit with the third of the Rogerian conditions, congruence (Rogers, 1957). The low expression of the critical/negativistic style (NT) indicates a calm temperament leading to equanimity. This attitude would be helpful in situations when both sides have to endure minimal progress or even setbacks that should not be the target of criticism or negativity. Instead, patients should be encouraged in such situations and guided with optimism. However, they should not be divested by psychotherapists of conducting their own therapeutic tasks. In this respect the low levels of the unselfish/self-sacrificing (SL) style could be interpreted. High scores of this style would reveal exaggerated helpfulness and social engagement with high degrees of self-sacrificing behavior. Schmidbauer (1977) most likely had this personality style in mind when writing the book on “The helpless helper.” Our psychotherapists, however, were below average on the SL scale. We had already found this in a previous study of German-speaking practitioners of hypnosis and hypnotherapy (Peter et al., 2012), interpreting the low level of the SL style as a specific product of professional socialization: psychotherapy’s primary purpose is to help patients helping themselves. Motivating patients requires a confident and active attitude. The self-critical style (SU) and its corresponding pathological expression, the avoidant personality disorder, describe people who are too sensitive to criticism, which does not apply to our psychotherapists. A passive attitude was also not found, which is represented by the passive/depressive style (DP), and is, in its pathological manifestation, accompanied by depression and feelings of worthlessness and inadequacy. Our psychotherapists are neither passive nor depressed; rather, they are obviously convinced of their self-efficacy. However, this confident attitude is not an assertion of ruthless, abusive, or humiliating behavior, as the psychotherapists also expressed low levels of the assertive/antisocial style (AS).

Finally, our psychotherapists generally showed low expression of the ST style compared to the normative sample. The intuitive/schizotypal style (ST) is characterized by a particular sensitivity to the mystery of events lacking logical explanation. In the extreme, it relates to the belief in clairvoyance and telepathy. The low ST values of our psychotherapists suggest a particular rational and enlightened perspective.

In our opinion this *second group* of personality styles, in which our sample of psychotherapists still differed significantly from the normative sample, is also indicative of their high social skills with regard to their profession. These personality styles could be considered to be *helpful* throughout the course of psychotherapy (e.g., being self-confident, but not aggressive, and being neither dependent nor too overly helpful).

In addition one could look at this second group of personality styles from a DSM-5 perspective. While the first group, in which the psychotherapists differed to a large extent from the norm, consisted of four personality styles that, if overly expressed, would seriously hamper the building of a trusting relationship (all of them pointing in their extreme form to personality disorders from Clusters A: “odd, eccentric,” and Cluster B: “dramatic, emotional”), some styles from the second group are, to some extent, helpful for relating to patients. Two of them, loyal/dependent (AB) and self-critical/avoidant (SU), as mentioned above, point in their extreme form to Cluster C personality disorders: “anxious, fearful, and avoidant.” Three more styles, passive/depressive (DP), critical/negativistic (NT), and the unselfish/self-sacrificing (SL) style, also would fit best to this group. Our psychotherapists were below the norms regarding these styles and are able to act appropriately (Hill, 2009), but they were so to a lesser extent, and are therefore able to engage in a compassionate and empathetic way with their patients. Moreover they are free of any pathological neuroticism, since four of the just mentioned styles were shown to be positively correlated with NEO-FFI neuroticism scale (Kuhl and Kazén, 2009); in other words, our psychotherapists were not anxious, avoidant, dependent or passive-aggressive. Finding only small to medium differences from the norm in the charming/histrionic (HI) style and no or small differences for the optimistic/rhapsodic (RH) style was somehow astonishing, as we had expected some degree of cheerfulness and optimism beyond the normative mean in our sample (Peter et al., 2012).

### Differences with Regard to Gender

To gauge the relevance of our findings, we should discuss them also in terms of gender. In our study, female psychotherapists differed from their male colleagues in seven styles with small and moderate effect sizes (albeit only three of them are actually pertinent to our research on psychotherapists, as we will see below; see **Figure 2**). It is noteworthy that in two of the four essential basic variables, the reserved/schizoid (SZ) and the ambitious/narcissistic (NA) styles, female therapists had significantly lower values than male therapists. That is, compared to men, they are to the emotional experience of their patients and are better able to put their own person in the background. This corresponds to their lower level, in comparison to men, of the assertive/antisocial (AS) style. Women are obviously more careful to not hurt others. It is fitting, then, that they were found to be more unselfish/self-sacrificing (SL) and self-critical/avoidant (SU), as well as more charming/histrionic (HI) and optimistic/rhapsodic (RH).

As mentioned previously, among the whole sample of psychotherapists, one style (HI) yielded a very small difference and two styles (RH and ZW) showed no difference from the norm. It is now evident that men differed remarkably from their female colleagues in two of them: they were significantly less charming/histrionic (HI) and optimistic/rhapsodic (RH).

– The optimistic/rhapsodic style (RH) is related to the charming/histrionic style (HI) and is characterized by a very positive attitude toward life, which makes it possible to bring out good aspects out of negative experiences.

In our opinion, sustained optimism is a *desirable* quality to have, especially in difficult psychotherapeutic situations when, for example in so-called impasses, patients have lost hope and psychotherapists have to step in. So we were surprised to find it expressed by female psychotherapists only, and only to a normal degree in relation to the normative sample.

Several of these gender differences were previously found in the normative sample of Kuhl and Kazén (2009), namely the SZ, ST, SU, AS and HI styles (see the “empty” arrows in **Figure 2**). These general gender-specific personality styles are integrated into the personality characteristics of psychotherapists, of which females make up two-thirds of the profession, and thus determine parts of the therapist variable. Beyond these general gender differences, female psychotherapists differed specifically from their male colleagues in three personality styles: they were less ambitious/narcissistic (NA), but more unselfish/self-sacrificing (SL) and more optimistic/rhapsodic (RH) (see the bold arrows in **Figure 2**). Moreover, it is interesting to note the gender differences that were found in the normative sample but not in our sample of psychotherapists. Specifically: women in general were found to be less willful/paranoid (PN), more loyal/dependent (AB), and above all less critical/negativistic (NT) than men, the latter with a medium effect, but this was not the case in our sample of psychotherapists, in which there were no significant gender differences regarding these three styles. We interpret this as a true product of psychotherapeutic socialization: Because both genders were below the averages of the normative sample, one must conclude that male psychotherapists have aligned their willful/paranoid (PN) and critical/negativistic (NT) styles to their female colleagues (compare the normal arrows above the profile lines in **Figure 2**; cf. **Table 6**).

Can one derive qualitative information for the therapist variable? Does all this perhaps mean that women are better psychotherapists? The answer should be yes if one is convinced that the personality styles identified by us are generally *necessary and helpful* for psychotherapeutic practice. These are especially found in female therapists. But should they be found in all psychotherapists? It is tautological to say that specific personality styles that are present specifically in women, are necessary and helpful for psychotherapy when two-thirds of those sampled, who have these personality characteristics, are female. Also, in a meta-analysis of 64 studies from the years 1930 to 2000, Bowman et al. (2001) found that the therapist’s gender has little overall effect on psychotherapy outcome (see also Lambert, 2016). So we can only conclude the following: women with these personality profiles, as found in our research, characterize the general personality profile of the psychological psychotherapist in German-speaking countries.

Do our results reflect a gender or culture-specific phenomenon? We think, the basic characteristics we have described as *necessary* for a good working alliance – low levels of willful/paranoid (PN), spontaneous/borderline (BL), ambitious/narcissistic (NA), and reserved/schizoid (SZ) – correspond well with two of the basic variables promoted by Rogers (1957) 50 years ago, i.e., unconditional positive regard and accurate empathy. These qualities, which were introduced rather explicitly by client-centered psychotherapy, have obviously become the basis of most psychotherapists, at least those with psychological training (because our sample included few with medical training, we cannot make this distinction). Since at least the late 1960s, the humanistic approach, which now seems to determine the modern therapeutic relationship, influenced gradually all main psychotherapy approaches. Consider for example that behavior therapy neglected the therapist variables for a long period of time (Margraf, 1996) and viewed the therapeutic relationship in terms of a quasi-pedagogical student-teacher or doctor-patient relationship, in which an insightful patient was amenable to their therapist’s rational considerations (Jaeggi, 1989), neglecting the influence of the psychotherapist’s personality in the therapeutic alliance. The patient entered into a “friendly submission” (DeVoge and Beck, 1978) and was accordingly more compliant to therapeutic instructions. This attitude has fundamentally changed at least since Beck et al. (1979) and other developments in cognitive-behavioral psychotherapies (Gilbert and Leahy, 2007), and our data seem to support that notion.

### Limitations

To conclude, we would like to emphasize how surprised we were as to how well the collected data coincided with our image of psychotherapists and their personalities. The personality styles we found are those in which the first author was socialized as a psychotherapist and has passed on as a teacher and supervisor to younger colleagues. Of course, this suggests the presence of an experimenter effect (Rosenthal, 1966), which we will consider regarding the interpretation of the data. However, for data collection, this can be ruled out because the selection of the three e-mail portals was based on a pragmatic criterion: each gave easy access to e-mail addresses of licensed therapists in each respective country. However, hardly any medical psychotherapists were contacted. Furthermore, the fact that only those with valid e-mail addresses were informed about our survey suggests, in theory, a systematic pre-selection process insofar that younger, more internet-savvy psychotherapists were successfully reached. But, the average age of participants and the proportion of female participants coincided well with samples from previous German studies that recruited participants by regular mail, such as Hessel et al. (2006), with an average age of 47 years and a 66% female participation rate, or Vangermain and Brauchle (2013), with an average age of 51 years and a 68% female participation rate. At the utmost, in our study respondents were slightly older and more likely to be female, but this corresponds roughly to the actual gender ratio in this profession. The 22% response rate of our study was lower than the response rate of approximately 35% of the aforementioned studies but well above the 9% response rate of, e.g., Grünberger and Laireiter (2014), who also used e-mail for recruitment. Basically, we deem our sample representative for psychotherapists of the three German speaking countries with respect to age and gender, and with 1,027 participants it is a large enough sample, to make up for the rather low response rate.

As our sample was representative for psychotherapists, it differed considerably from the normative sample for the PSDI-S. The latter was – for obvious reasons – gender-balanced and on average younger, encompassing all age groups from 12 to 82 years. And although we do not have data on the educational level of the normative sample we assume they were also less educated as our academic sample. When choosing our analytic strategy, we had to decide whether to use only parts of the normative sample (dismissing about 30% of its participants) so that it would correspond to the age range of the psychotherapist sample, or use the entire normative sample as basis for the computation of *T*-scores. We chose the latter because we wanted to compare the psychotherapists to a norm based on as broad of a sample out of the general population as possible. As a consequence, we cannot rule out for certain that some of the psychotherapists’ differences from the PSDI-S norms are actually the result of them being older, more educated, and having a greater proportion of female participants than the sample on which these norms are based on.

We also do not know whether self-selection bias affected the results in the sense that those with functional personality styles or simply the especially helpful ones, were more likely to respond to the participation request. Given the actual values (average for women and even below average for men) for the unselfish/self-sacrificing style (SL), this is unlikely though still possible. In addition, we do not know whether social desirability or other response tendencies played a role, but the fact that the internet questionnaire was anonymous at least allowed participants to answer truthfully.

In other words, we can only infer about how the participants saw themselves or perhaps wanted to be seen, and not as they really are, but this really applies to all statements that are collected by self-report instruments. Of importance to our study is that the participating psychotherapists were able to use their clinical knowledge to evaluate the items and their meaning more than a normative sample could. That could mean that the significantly lower average values, compared to the norm, may have been the result of the participants’ motivation to distance themselves distinctly from personality disorders, especially those from Cluster A (odd, eccentric, i.e., paranoid and schizoid personality disorders) and B (dramatic, emotional, i.e., narcissistic and borderline personality disorders). In terms of social desirability, perhaps the responding psychotherapists did not in any way want to be connected to mental disorders that would seriously impair their ability to build a therapeutic relationship with their clients.

Even if such response tendencies played a role, we believe that psychotherapists actually are of above-average functionality when they act as a professional. They can obviously switch between private and professional attitudes: Heinonen and Orlinsky (2013) found psychotherapists differ in their relational manner at home with family or partners and in their office with patients, which is, in our opinion, also a sign of soundness. Up to now, few studies have dealt with psychotherapists’ functionality in private relationships (Heinonen and Orlinsky, 2013) or their mental health status in general (von Sydow, 2014). In any case, we cannot determine whether these characteristics are genuine, primary dispositions, just “vocational stances” or true personality formations. We suspect the latter. Using NEO-FFI-items among others Grünberger and Laireiter (2014) asked 152 Austrian psychotherapists, how their psychotherapeutic practice had impacted their personal lives. The psychotherapists reported, compared to the beginning of their career, an increase in tolerance and openness and a decrease in neuroticism. They also experienced being more sensitive and confident. Similar findings were reported by Orlinsky and Rønnestad (2005) in their comprehensive review “How Psychotherapists Develop.” In a previous study on personality styles of psychology majors compared to majors in more genuine natural sciences (i.e., so-called STEM-fields), we found very few differences between the departments (Bochter et al., 2014). Psychology students were a little less reserved/schizoid (SZ) as those studying subjects such as math, computer science, biology, or engineering, but, rather surprisingly, they were still significantly higher than the norm, unlike our sample of psychotherapists. Overall, the psychology students differed little from the norm, and even less so than their fellow students from the STEM-fields. Taken together, we believe that psychotherapists, in the process of their professional socialization, learn to lay aside less functional personality and relationship styles, which is then reflected in the results of our investigation.

Of course, due to the nature of our internet survey, we have no data about the actual effectiveness and efficiency of our psychotherapists. We assume that the below-average values of certain personality styles are necessary for building therapeutic relationships and helpful for intervening effectively. In the grouping of those relationship and intervention skills postulated by us, we followed the ranking of the effect sizes resulting from the comparisons to the norm. One might criticize that this was a *post hoc* classification and circular reasoning at that: we assume that these personality styles are helpful for psychotherapists because we have found them in the psychotherapists we have studied. As already mentioned, the results match well with our prototype of the “good psychotherapist,” so interpretation bias could have influenced our discussion of the results.

Taken together, the main limitations of this study are rooted in the interpretation of our data, which may be based on our own psychological background and, in the case of the first author, psychotherapeutic socialization. Another major limitation is related to the chosen methodology, as we did not control for demographics in our comparisons. We also do not know how social desirability influenced the response tendencies of the psychotherapists. Future studies might take a closer look at the personality and interactional styles of psychotherapists, perhaps with the help of observational data and clinical ratings in addition to self-report data and in relation to psychotherapy outcome. Furthermore, these results only apply to non-medical psychotherapists working in German-speaking countries. All in all, we consider our work as exploratory, wanting to generate hypotheses and results that are now open for replication.

## Conclusion

Our study is one of the very few on personality traits of psychotherapists. We demonstrated that (1) the personality profiles of psychotherapists from three German-speaking countries are very similar. (2) However, they differ significantly from the personality profiles of the general population. (3) These differences are meaningful with respect to the profession of the psychotherapists: According to our interpretation, these personality profiles contribute to the relationship skills of psychotherapists. (4) Female psychotherapists present these personality styles to a larger extent than their male colleagues.

## Ethics Statement

In accordance with the Declaration of Helsinki and local legislation, no formal institutional approval was sought because of the very nature of the study (online survey targeting adults and using a transparent, non-offending personality questionnaire): Psychotherapists were contacted by e-mail addresses retrieved from three public psychotherapy search portals and informed openly about the aim and purpose of the study. They filled in the questionnaire anonymously and received no compensation for participation. Therefore, no additional written consent was sought other than the consent expressed by participating.

## Author Contributions

The study was conceptualized by BP who also organized data collection and study setup and wrote most of the manuscript. EB performed the statistical analysis and contributed substantially to writing the method and results section. MH contributed substantially to data analysis and writing and editing the manuscript. MR was mainly responsible for data collection and preparation. MK provided all the material of the PSDI including syntax and the data of the normative sample. All authors were active in revising the manuscript.

## Conflict of Interest Statement

The authors declare that the research was conducted in the absence of any commercial or financial relationships that could be construed as a potential conflict of interest.

## Footnotes

1 A survey conducted by the German Federal Medical Association from 2003 reported that females make up exactly 66.6% of 12,249 psychological psychotherapists and 63.5% of 3,606 medical psychotherapists. The website of the Austrian Ministry of Health gives a 72% proportion of females among 8,541 psychotherapists. The respective website of the Federation of Swiss Psychologists counts 69% females among 2,423 psychotherapists.

2 This is the result of specific legislation in Austria, which permits non-psychologists and non-physicians to go through professional training in psychotherapy.

3 deutschepsychotherapeutenvereinigung.de/nc/patienten/psychotherapeutensuche/

4 psychotherapie.ehealth.gv.at

5 https://www.psychologie.ch/psychologie/psychologinnen-verzeichnis/psychologinnen-verzeichnis/

6 *p* = 0.025/14 = 0.0018.
